# N‐Heterocyclic Carbenes Carrying Weakly Coordinating Anions

**DOI:** 10.1002/chem.202200530

**Published:** 2022-04-27

**Authors:** Luong Phong Ho, Matthias Tamm

**Affiliations:** ^1^ Institut für Anorganische und Analytische Chemie Technische Universität Braunschweig Hagenring 30 38106 Braunschweig Germany

**Keywords:** catalysis, main group element chemistry, N-heterocyclic carbene, transition metal chemistry, weakly coordinating anions

## Abstract

In this Concept article we provide a brief overview of the design and preparation of N‐heterocyclic carbenes carrying weakly coordinating anions (WCA‐NHCs). The anionic charge in these ligand systems is located on an exocyclic group, for example, B(C_6_F_5_)_3_, tethered to the backbone of the imidazole ring, thus resembling a weakly coordinating moiety. With the general guiding principle behind the application of WCA‐NHCs being the conversion of otherwise cationic NHC complexes into their overall neutral congeners, numerous transition metal as well as main group element complexes were isolated during the last decade, which are summarized herein.

## Graphical abstract

**Piggyback**: N‐heterocyclic carbenes carrying weakly coordinating anions (WCA‐NHCs) are an important and versatile ligand class used in transition metal as well as main group element chemistry. Bearing a negative charge in the backbone of the heterocycle, these carbenes can convert otherwise cationic NHC complexes into their overall neutral congeners. Aspects such as their development, syntheses, reactivity and possible future applications are discussed within this concept article.

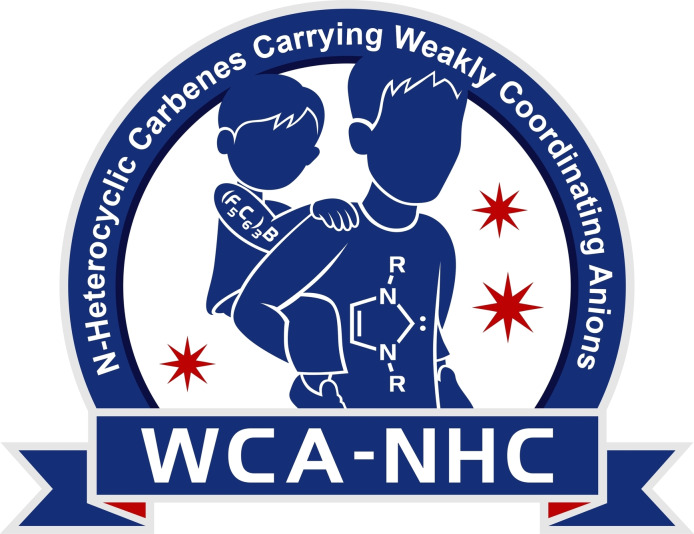

## Introduction

**Figure 1:**
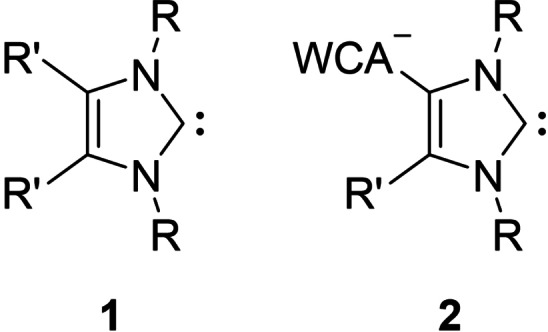
Left: N‐heterocyclic carbene (NHC). Right: N‐heterocyclic carbene carrying weakly coordinating anions (WCA‐NHC).

Emerging from Arduengo's pioneering work in isolating the first stable crystalline N‐heterocyclic carbene (NHC, **1**, Figure 1) in 1991,^[1]^ this ligand class became rapidly one of the cornerstones in modern organometallic chemistry,[^2^, ^3^] with applications in both transition metal^[4]^ as well as main group element chemistry.[^5^, ^6^, ^7^, ^8^, ^9^, ^10^] These cyclic neutral compounds, incorporating a divalent carbon atom with an electron sextet, and additionally at least one nitrogen atom in the α position, have strong σ‐donating capabilities as the adjacent nitrogen atoms can conduct π‐electron density donation from their lone pairs into the *p*‐orbital of the carbene carbon atom (+*M*‐effect) and thereby increase the HOMO–LUMO gap. This in turn results in the predominant singlet state of NHCs, with both its nonbinding electrons occupying the *sp* ^2^‐hybrid orbital. Additionally, with the population of this orbital located in the N−C−N bond plane, the stability is further increased by the σ‐inductive withdrawing effects of the nitrogen atoms (‐*I*‐effect).[^2^, ^11^]

The steric and electronic properties of NHCs can be readily modified by altering the nitrogen‐ or backbone substituents to meet the desired requirements for their intended use. For instance, one of these variations consists in the tethering of an anionic moiety to the otherwise neutral NHC ligand, and the chemistry involving these resulting anionic N‐heterocyclic carbenes has witnessed considerable interest throughout the last decade.^[12]^ Anionic NHCs can be classified into two different groups: one where the anionic charge is in π‐conjugation with the heterocycle and one where it is not π‐conjugated, with the latter type, providing zwitterionic character upon coordination to a transition metal or a main group element. The N‐tethering of the anionic charge often enables a bidentate binding mode of the ligand via the carbene carbon atom as well as the anionic moiety,^[13]^ whereas the backbone functionalization with an anionic moiety provides monodentate ligands, with charge separation from the usually cationic (metal) fragment in the corresponding complexes. The non‐delocalized anionic charge can be allocated on moieties comprised of transition metals, four‐coordinated group 13 elements, carborane groups, three‐ or five‐coordinated group 14 elements or even carboxylate groups.^[12]^ Not only simple anionic moieties can be attached, but also groups resembling a weakly coordinating moiety (WCA), for example B(C_6_F_5_)_3_, and therefore, these specific type of anionic NHCs were termed WCA‐NHC (**2**).

The concept of converting otherwise cationic NHC transition metal or main group element complexes into neutral and zwitterionic analogues can be recognized as one of the general guiding principles involving the chemistry of anionic NHCs. This becomes clear, for example, with NHC‐AuCl complexes,^[14]^ which have to be activated with a silver(I) salt in order to generate the corresponding cationic gold(I) complex as the catalytically active species in the presence of a weakly coordinating anion. The use of a WCA‐NHC (see below) directly eliminates the need to remove the chloride from the gold(I) atom with auxiliary salts and additionally, as the WCA is tethered to the NHC, the anion is less likely to interfere with the catalytically active site.^[15]^ Furthermore and in contrast to their cationic counterparts, these zwitterionic complexes have advantageous solubilities in low‐polarity media and hence, often display superior catalytic performances.^[16]^

In recent years, our group has significantly advanced the chemistry concerning N‐heterocyclic carbenes carrying weakly coordinating anions, not only encompassing the d‐block, but also pushed forward with these ligand systems into the main group element domain. Therefore, with this concept paper, we want to provide a short synopsis about the latest developments regarding the WCA‐NHC chemistry, with a focus on our own contribution to this rapidly evolving field and will conclude with a brief outlook on what is potentially to come in the upcoming decade.

## State of the Art

### Development and preparation of WCA‐NHCs

**Scheme 1:**
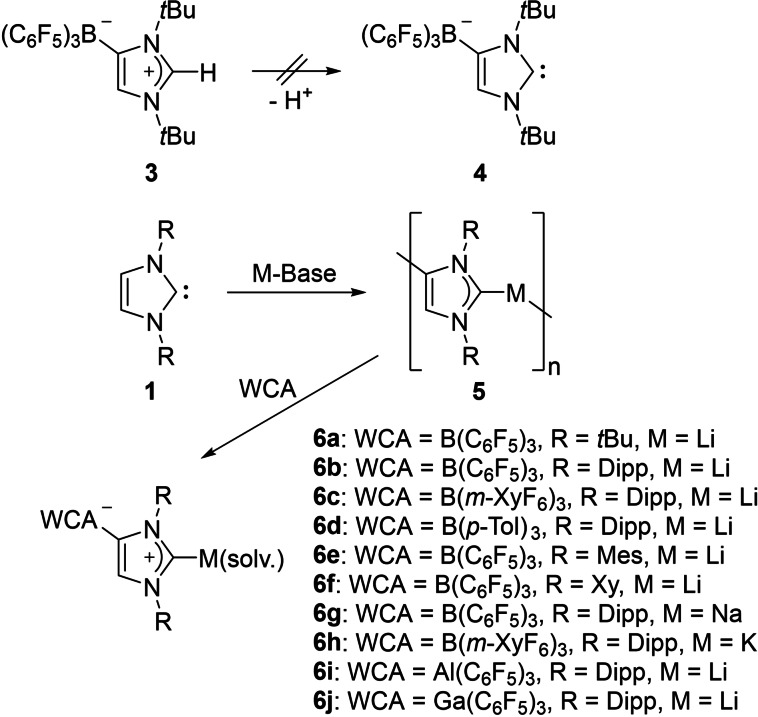
Synthesis of N‐heterocyclic carbenes carrying weakly coordinating anions.

In 2008, our group discovered that the frustrated Lewis pair (FLP) system consisting of I*t*Bu (I*t*Bu=1,3‐di‐*tert*‐butylimidazolin‐2‐ylidene) and B(C_6_F_5_)_3_ undergoes a self‐deactivation reaction in toluene over a time span of two hours at room temperature, when no small molecule, for example H_2_, is offered to the FLP to split. Apparently, an abnormal NHC adduct **3** (Scheme 1) was obtained under these conditions, where one proton of the imidazole backbone migrates to the C2 carbene carbon atom, resulting in the B(C_6_F_5_)_3_ now attached to the C4 position.^[17]^ Attempts to deprotonate this species, which can be regarded as the protonated version of the corresponding anionic carbene WCA‐I*t*Bu **4**, remained so far unsuccessful. The C−F activation at the arene substituents might be favored, as strong bases such as LDA or *n*BuLi are required to achieve potential deprotonation of the highly basic C2 position.

**Figure 2:**
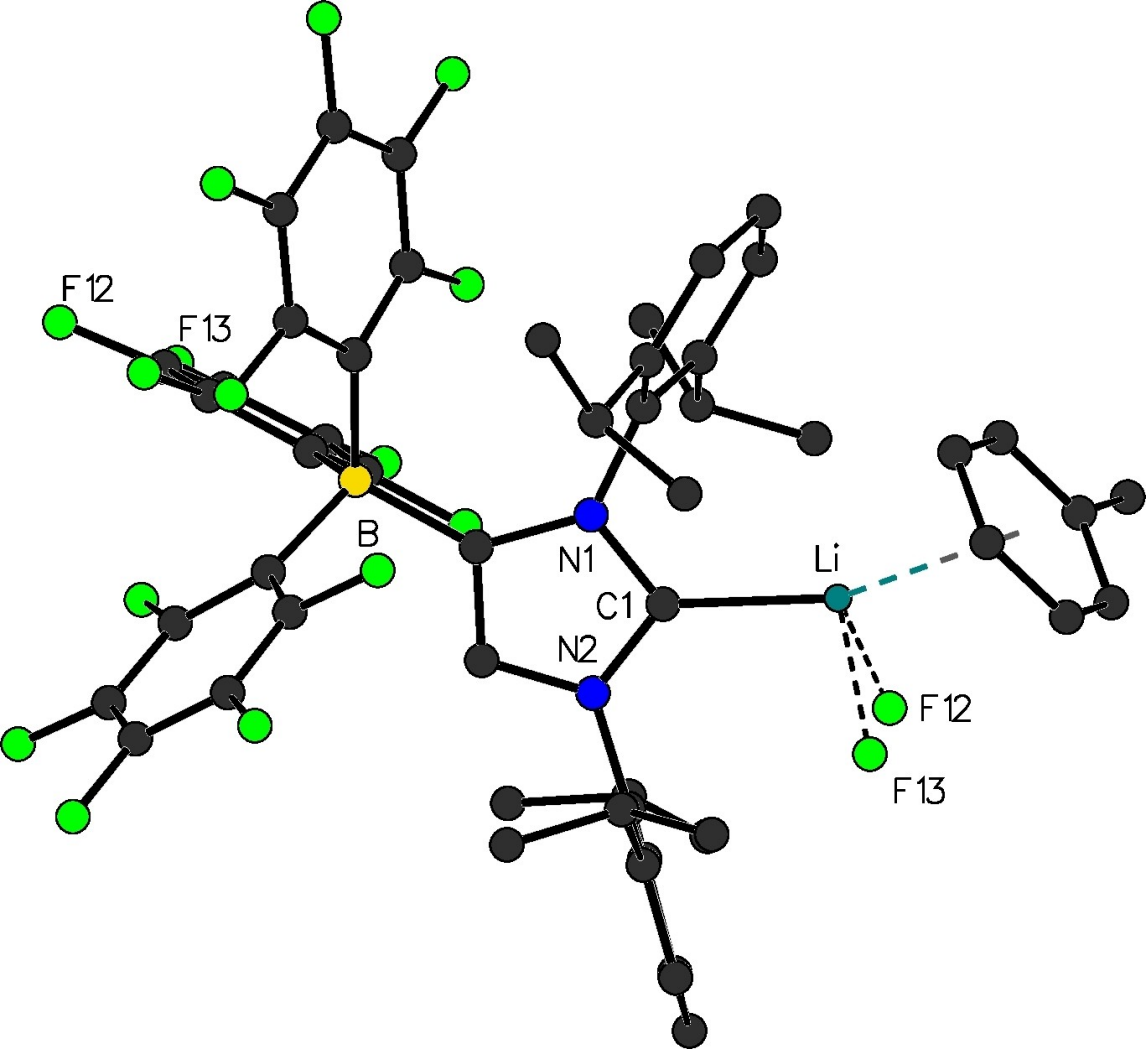
Molecular structure of [(F_5_C_6_)_3_B‐IDipp]Li(toluene) (**6** **b**).

In 2010 Robinson and co‐workers developed a reliable synthetic protocol to deprotonate an N‐heterocyclic carbene at the C4 position with *n*BuLi, giving access to anionic “dicarbene” **5**.^[18]^ In the solid state, a polymeric structure was found for this lithiated species, and it can be subsequently reacted with a variety of group 13 Lewis acids, such as BEt_3_, AlMe_3_ or Ga(CH_2_SiMe_3_)_3_,[^18^, ^19^] to provide abnormal adducts, where the now anionic group 13 moiety is attached to the C4 position of the NHC and a lithium cation at the C2 position. Adapting this strategy in 2012, our group was able to deprotonate several differently nitrogen‐substituted NHCs in *n*‐hexane solution with *n*BuLi, allowing the anionic dicarbenes **5** to be isolated as white precipitates. The subsequent reaction of **5** with B(C_6_F_5_)_3_ in toluene then affords the lithium salts of the corresponding N‐heterocyclic carbenes carrying weakly coordinating anions [(F_5_C_6_)_3_B‐NHC]Li(thf)_3_ (**6** **a**: NHC=I*t*Bu; **6** **b**: NHC=IDipp) after recrystallization from THF.^[15]^ One year later in 2013, the synthetic procedure to isolate these WCA‐NHCs was refined into a convenient one‐pot procedure. The NHC is dissolved in toluene and upon addition of *n*BuLi, a significant increase in viscosity of the carbene solution can be observed, accompanied by a distinct color change from yellow to deep orange. After stirring overnight, a tris(aryl)borane can be added to the orange gel, resulting in a swift decrease of viscosity, with a colorless precipitate forming from the resulting pale‐yellow solution after a few hours. This precipitate can be collected via filtration, washed with toluene and dried in vacuo to give the toluene solvates [WCA‐NHC]Li(toluene) (**6** **a**–**f**, WCA=B(C_6_F_5_)_3_, B(m‐XyF_6_)_3_, B(p‐Tol)_3_; NHC=I*t*Bu, IDipp, IMes, IXy; m‐XyF_6_=3,5‐bis(trifluoromethyl)phenyl; p‐Tol=4‐methylphenyl; IDipp=1,3‐bis(2,6‐diisopropylphenyl)imidazolin‐2‐ylidene; IMes=1,3‐bis(2,4,6‐trimethylphenyl)imidazolin‐2‐ylidene; IXy=1,3‐bis(2,4‐dimethylphenyl)imidazolin‐2‐ylidene; see Figure 2 for a representation of **6** **b**) as highly moisture sensitive solids in good yields (70–99 %).[^20^, ^21^] With this method, WCA‐NHCs were now easily accessible and affordable in large scales, which jump‐started their broad range application in transition‐metal chemistry as well as main‐group‐element chemistry.

Recently, it was also possible to isolate the heavier alkali metal derivatives, using an appropriate combination of strong bases. For the sodium carbene [(F_5_C_6_)_3_B‐IDipp]Na(thf)_3_ (**6** **g**), IDipp was deprotonated with a mixture of NaHMDS/*n*BuLi in Et_2_O and then treated with B(C_6_F_5_)_3_ in toluene, followed by recrystallization from THF (88 % yield). Likewise, deprotonation of IDipp with KHMDS/*n*BuLi and subsequent reaction with B(m‐XyF_6_)_3_ yielded [(m‐XyF_6_)_3_B‐IDipp]K(thf)_3_ (**6** **h**, 60 % yield).^[22]^ Furthermore, lithium salts of WCA‐NHCs containing aluminate‐ or gallate‐functionalized backbones were also prepared in our group. Following the same synthetic strategy as described above, the reaction of the dicarbene **5** with Al(C_6_F_5_)_3_ or Ga(C_6_F_5_)_3_, furnished the complexes [(F_5_C_6_)_3_E‐IDipp]Li(thf)_2_ (**6** **i**: E=Al; **6** **j**: E=Ga) in 45 and 79 % yields, respectively.^[23]^

**Figure 3:**
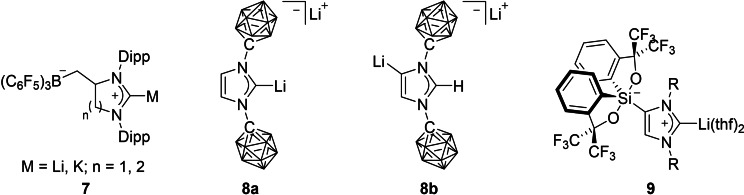
Examples for other WCA‐NHCs.

In 2014, Aldridge and co‐workers presented an alternative route for the incorporation of the weakly coordinating anion to an N‐heterocyclic carbene. They discovered that the acyclic system *N*,*N*′‐bis‐(diisopropylphenyl)‐*N*‐allylformamidine undergoes ring closure in the presence of Lewis acids, such as B(C_6_F_5_)_3_, B(*m*‐XyF_6_)_3_ or Al(OC(CF_3_)_3_)_3_, to form a heterocycle featuring a WCA group. Surprisingly, this ring closure appears to be highly regioselective, as only the *exo* product was obtained, producing protonated WCA‐NHCs, where the WCA group is tethered to the heterocycle via a CH_2_ linker. Unlike the protonated WCA‐NHCs developed by our group, these imidazolium salts can be deprotonated with sufficiently strong bases, for example, *n*BuLi or KHMDS, generating the corresponding alkali metal complexes **7** (Figure 3). The different behavior might be reasoned by the electronic separation of the borate moiety from the imidazole plane enforced by the saturated CH_2_ linker, making them more related to the parent imidazolium salt in terms of basicity.[^24^, ^25^]

A completely different approach to tether a weakly coordinating anion to an NHC was introduced by Lavallo and co‐workers in 2014, where they fused NHCs with carborane anions. Starting from a carba‐*closo*‐dodecaboranyl amine anion, a double condensation reaction with glyoxal was performed to afford a dianionic diamine, which was subsequently treated with paraformaldehyde, activated with HCl, to induce the ring closure. The deprotonation of the resulting anionic imidazolium salt bearing two icosahedral carba‐*closo*‐dodecaborate groups (CB_11_H_11_) with LiHMDS occurs solely at the C2 position to cleanly afford the normal dianionic NHC **8** **a**, whereas the backbone deprotonated dianionic NHC **8** **b** is selectively obtained with LDA at −78 °C.^[26]^ His group was also able to isolate unsymmetrical N‐carboranyl NHCs, whereby the heterocyclic interconversion of a mesityl substituted oxazolinium cation, via reaction with a carba‐*closo*‐dodecaboranyl amine anion, was performed. The corresponding anionic N‐carboranyl NHCs were then accessed by deprotonation of aforementioned imidazolium salt with LiHMDS or KHMDS, respectively.^[27]^ The latter strategy was also applied to obtain a unsymmetrical mono‐*nido*‐carboranyl imidazolium zwitterion, which was deprotonated with NaHMDS, to yield a N‐dicarbollide NHC dianion.^[28]^

In 2018, Fensterbank and co‐workers reported the isolation of anionic NHCs bearing a weakly coordinating siliconate moiety. The reaction of a sterically demanding spirosilane with IDipp or I*t*Bu in toluene, produced abnormal adducts, where the silane attached itself to the C4 position of the imidazole rings and the C2 position now being protonated. It may be noteworthy to mention that this species, and to be more specific the siliconate moiety, appears to be exceptionally robust, as purification over silica could be performed without decomposition of this WCA group. The deprotonation of the imidazolium salt was cleanly achieved with *n*BuLi, affording the corresponding lithium salts **9**. ^[29]^

### WCA‐NHCs in Transition Metal Chemistry

**Figure 4:**
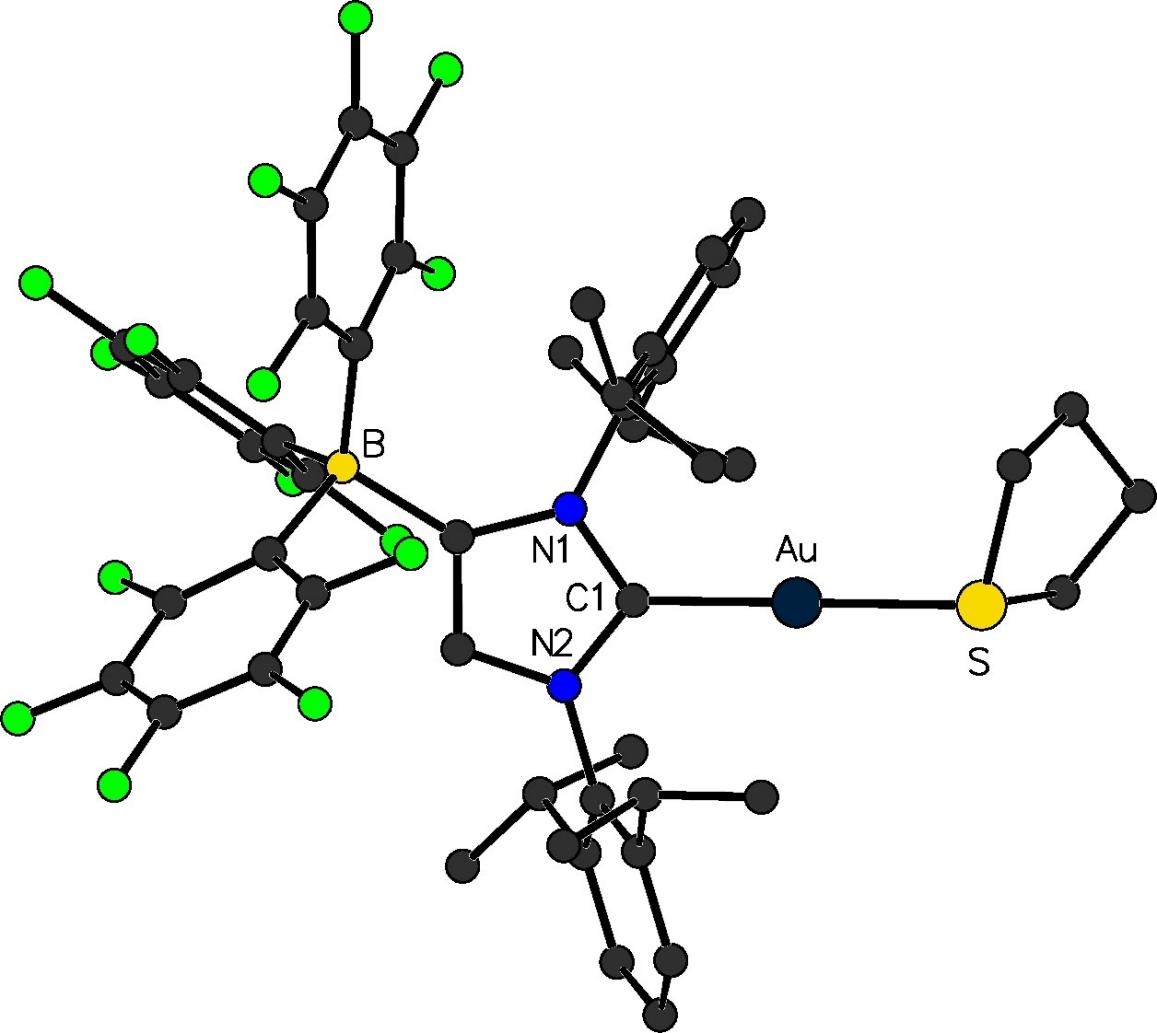
Molecular structure of [(F_5_C_6_)_3_B‐IDipp]Au(tht) (**10** **b**⋅tht).

**Figure 5:**
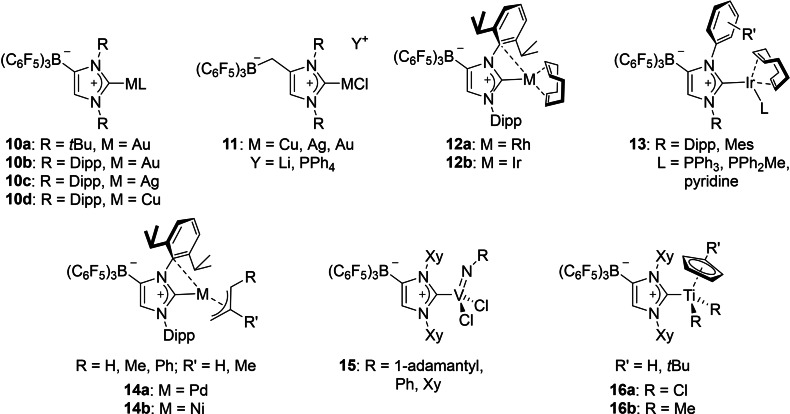
Selected examples for WCA‐NHC transition metal complexes.

The lithium salts of the above discussed N‐heterocyclic carbenes carrying weakly coordinating anions were readily applied in transmetallation reactions, where the [WCA‐NHC]Li(solv.) species act as anionic carbene transfer reagents. With suitable transition metal halide precursors, lithium halide elimination can be facilitated to yield overall neutrally charged transition metal complexes. Among the first transition metal complexes bearing a WCA‐NHC ligand were gold(I) complexes, isolated by our group in 2012. [(F_5_C_6_)_3_B‐NHC]Li(thf)_2_ (**6** **a**: NHC=I*t*Bu; **6** **b**: NHC=IDipp) was employed in a salt metathesis reaction with (L)AuCl (L=tht=tetrahydrothiophene, L=PPh_3_), to afford the corresponding complexes [(F_5_C_6_)_3_B‐NHC]AuPPh_3_ (**10** **a**⋅PPh_3_: NHC=I*t*Bu; **10** **b**⋅PPh_3_: NHC=IDipp) and [(F_5_C_6_)_3_B‐IDipp]Au(tht) (**10** **b**⋅tht, Figures 4 and 5) as colorless, crystalline solids after separation from LiCl. As already briefly addressed in the introduction (see above), for catalytic applications, for example, the cyclization and isomerization of enynes by skeletal rearrangement, regular NHC‐AuCl complexes require auxiliary chloride scavengers, usually silver(I) salts AgX (X=PF_6_, BF_4_ etc.), to be activated. It is necessary to generate the corresponding cationic gold(I) complex, which then acts as the catalytically active species in the presence of a WCA. With the use of the WCA‐NHC, the cationic gold(I) fragment can now be stabilized directly with an anionic carbene and without the presence of the chloride in the coordination sphere of the metal. This eliminates the need for the addition of AgX for activation, since the complex **10** **b**⋅tht can be used directly as a stand‐alone catalyst for these model reactions, performing well even in non‐polar solvents like toluene.^[15]^ Furthermore, the similar complex [(F_5_C_6_)_3_B‐IDipp]Au(SMe_2_) (**10** **b**⋅SMe_2_) was successfully employed as an efficient catalyst for the hydration of terminal and internal alkynes without the need for silver or acid additives, highlighting the value of the WCA‐NHC ligand in gold catalysis.^[30]^

The reaction of the lithium salt **6** **b** with CuCl or AgCl in THF does not result in the elimination of LiCl. Instead the copper complex [{(F_5_C_6_)_3_B‐IDipp}CuCl]Li(thf)_4_ was obtained, whereas with silver the bis(carbene) adduct [{(F_5_C_6_)_3_B‐IDipp}_2_Ag]Li(thf)_4_ was formed selectively, independent from the stoichiometry of the substrates used during the reaction. Changing the solvent to toluene facilitated LiCl elimination with chloro(triphenylphosphane)‐silver(I) and ‐copper(I) salts, yielding the desired complexes [(F_5_C_6_)_3_B‐IDipp]MPPh_3_ (**10** **c**⋅PPh_3_: M=Ag; **10** **d**⋅PPh_3_: M=Cu). Phosphane‐free WCA‐NHC copper(I) and silver(I) complexes were also prepared staring from CuCl and AgOTf, respectively. The reaction with **6** **b** in toluene gave rise to the toluene solvates [(F_5_C_6_)_3_B‐IDipp]M(toluene) (**10** **c**⋅toluene: M=Ag; **10** **d**⋅toluene: M=Cu), where one co‐crystallized toluene molecule coordinates to the coinage metal atom. The silver(I) complex was anticipated to act as a novel WCA‐NHC transmetallation reagent upon reaction with [(*η* ^6^‐*p*‐cymene)RuCl_2_(PPh_3_)], with the ultimate goal to prepare ruthenium(II) WCA‐NHC complexes for application in olefin metathesis. However, the resulting heterobimetallic complexes [(F_5_C_6_)_3_B‐IDipp]Ag(μ^2^‐X)_2_Ru(η^6^‐*p*‐cymene)(PPh_3_) (X=Cl, I) resisted silver halide elimination, regardless of the examined reaction conditions.^[31]^

Coinage metal complexes were also prepared with Aldridge's WCA‐NHCs, where the WCA moiety is linked via a CH_2_ linker to the heterocycle, but the complexes [{(F_5_C_6_)_3_BCH_2_‐SIDipp}MCl]Y (**11**, M=Cu, Ag, Au; Y=Li, PPh_4_; SIDipp=1,3‐bis(2,6‐diisopropyl)imidazolidin‐2‐ylidene) were not yet employed for any catalytic studies.^[25]^ Silver(I) and gold(I) complexes featuring Lavallo's carboranyl N‐heterocyclic carbenes were also realized. For instance, the lithium chloride elimination was accomplished via the reaction of the corresponding carboranyl NHC lithium salt, with (Me_2_S)AuCl in fluorobenzene^[32]^ and some of these derivatives were found to be highly active catalysts for the hydroamination of alkynes.^[33]^ Silver complexes of tethered N‐heterocyclic carbene‐carboranyl ligands have been prepared, starting from the protonated carbene and Ag_2_O in dichloromethane. The in vitro cytotoxicity of these complexes were examined against the colon cancer cells.^[34]^

**Figure 6:**
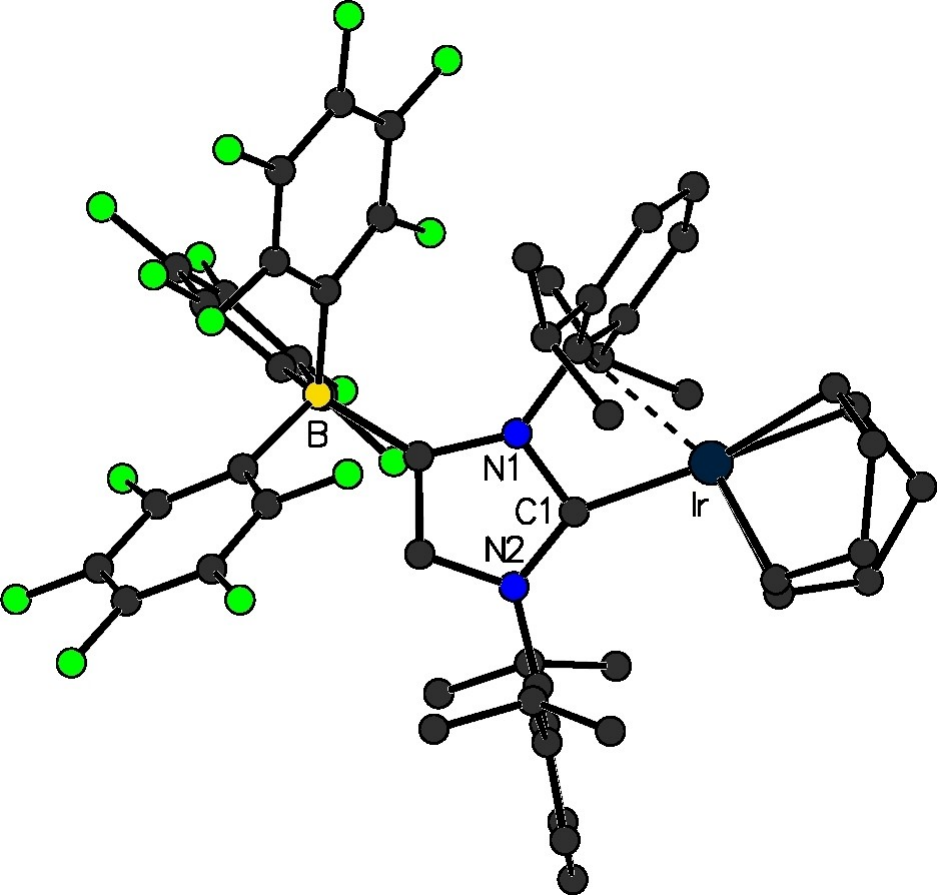
Molecular structure of [(F_5_C_6_)_3_B‐IDipp]Ir(COD) (**12** **b**).

Rhodium(I) and iridium(I) complexes with WCA‐NHC ligands were prepared by our group in 2013.^[20]^ The reaction of **6** **b** with dimeric [M(COD)Cl]_2_ (M=Rh, Ir; COD=cyclooctadiene), afforded, after separation of LiCl, the zwitterionic complexes [(F_5_C_6_)_3_B‐IDipp]M(COD) (**12** **a**: M=Rh; **12** **b**: M=Ir; Figure 6). Surprisingly, the metal atoms in these species exhibit an intramolecular interaction (π‐face donation) with the borate flanking N‐aryl groups of the carbene ligands (*syn*‐isomer). Accordingly, theoretical investigation revealed that the interaction on this side is energetically favored by 6–9 kcal mol^−1^ over the aryl group opposite to the borate moiety (*anti*‐isomer). Inspired by Crabtree's catalyst [Ir(COD)(py)(PCy_3_)][PF_6_], which is a highly active and selective hydrogenation catalyst, the complex [(F_5_C_6_)_3_B‐IDipp]Ir(COD) (among other [WCA‐NHC]Ir(COD) derivatives) was tested as a catalyst for the homogeneous hydrogenation of alkenes. High activity was observed in non‐polar solvents or in the neat alkene substrate, even with very low catalyst loadings.^[20]^ The complexes [(F_5_C_6_)_3_B‐NHC]Ir(COD) were further modified by addition of phosphanes or pyridine, to replace the metal–arene interaction. The resulting complexes [(F_5_C_6_)_3_B‐NHC]IrL(COD) (**13**, NHC=IDipp, IMes; L=PPh_3_, PPh_2_Me, pyridine) were employed for the catalytic hydrogen isotope exchange, revealing their suitability to promote efficient deuteration of a wide scope of substrates in non‐polar solvents such as cyclohexane.^[35]^ One iridium(I) cyclooctadiene complex bearing Adridge's CH_2_‐linked WCA‐NHC (see above) was also reported, however, no catalytic studies were conducted with this complex.^[24]^

In 2016, we reported the isolation and characterization of (allyl)palladium(II) complexes with N‐heterocyclic carbenes carrying a weakly coordinating borate moiety.^[36]^ The reaction of **6** **b** with [(*η* ^3^‐allyl)Pd(*μ*‐Cl)]_2_ (allyl=2‐propenyl, 2‐butenyl, 2‐methyl‐2‐propenyl, 1‐phenyl‐2‐propenyl) gave rise to chloropalladate complexes of the type [{(F_5_C_6_)_3_B‐IDipp}PdCl(*η* ^3^‐allyl)]Li(thf)_3_ if the reaction was performed in THF solution. In contrast, LiCl elimination was achieved in toluene solution, yielding [(F_5_C_6_)_3_B‐IDipp]Pd(*η* ^3^‐allyl) complexes **14** **a**, which again exhibit an intramolecular interaction with the N‐aryl groups of the carbene ligands flaking the borate moiety. In line with the theoretical investigations performed for the Rh(I) and Ir(I) systems, density functional theory (DFT) calculations confirmed the preference of the experimentally observed *syn*‐isomer by 3.5 kcal mol^−1^ over the *anti*‐isomer. We expected the reversibility of this metal‐arene interaction in these complexes, which might give access to catalytically active 14‐electron palladium species. Therefore, they were tested as catalysts for the Buchwald–Hartwig cross‐coupling of 4‐bromotoluene and morpholine, revealing their suitability to promote these reactions.^[36]^ Similar nickel(II) complexes were also accessible. Following the reaction of **6** **b** with [(*η* ^3^‐C_3_H_5_)Ni(*μ* ^2^‐Cl)]_2_ in THF, the chloronickelate complex [{(F_5_C_6_)_3_B‐IDipp}NiCl(*η* ^3^‐C_3_H_5_)]Li(thf)_3_ was obtained. The subsequent treatment of this complex with toluene facilitated the elimination of LiCl, providing [(F_5_C_6_)_3_B‐IDipp]Ni(*η* ^3^‐C_3_H_5_) (**14** **b**). In addition, we found that the vacant coordination site at the nickel(II) atom in complex **14** **b** can be saturated with strong σ‐donor ligands. Therefore, the addition of carbon monoxide or phosphanes furnished complexes of the general type [(F_5_C_6_)_3_B‐IDipp]PdL(*η* ^3^‐C_3_H_5_) (L=CO, PMe_3_, PMe_2_Ph, PMePh_2_).^[37]^

(Imido)vanadium(V) dichloride complexes containing an anionic N‐heterocyclic carbene ligand with a weakly coordinating borate moiety were established in 2016 by Nomura and co‐workers in cooperation with our group. The compounds [(F_5_C_6_)_3_B‐IXy]V(NR)Cl_2_ (**15**, R=1‐adamantyl, Ph, Xy) were prepared from the reaction of the lithium salt [(F_5_C_6_)_3_B‐IXy]Li(toluene) (**6** **f**) with the corresponding V(NR)Cl_3_. In the solid state‐structures, the orientation of the imido group bearing the adamantly substituent is in *syn*‐orientation, with respect to the borate moiety, whereas for the phenyl and xylyl derivatives, an *anti*‐orientation was found. π‐Stacking interactions were found between one C_6_F_5_ group and the neighboring xylyl nitrogen‐substituent. In contrast to the Rh(I) (**12** **a**), Ir(I) (**12** **b**), Pd(II) (**14** **a**) and Ni(II) (**14** **b**) complexes discussed above, intramolecular arene‐metal contacts were not observed. These vanadium(V) complexes exhibited remarkable catalytic activity for ethylene polymerization reactions in the presence of MAO or even with Al*i*Bu_3_, the latter usually being regarded as the less efficient Al co‐catalyst. These catalysts afforded linear polyethylene with unimodal molecular weight distribution, with the high performance ascribed to the stabilization of (formally) cationic alkyl species by the WCA‐NHC ligand.[^21^, ^38^] Furthermore, ethylene/norbonene co‐polymerization was achieved with these catalysts.^[39]^

Titanium half‐sandwich complexes bearing a WCA‐NHC were established in 2019. The straightforward reaction of [(F_5_C_6_)_3_B‐IXy]Li(toluene) (**6** **f**) with LTiCl_3_ (L=Cp, Cp’; Cp’=*t*BuC_5_H_4_) afforded the complexes [(F_5_C_6_)_3_B‐IXy]TiLCl_2_ (**16** **a**) as orange microcrystalline solids. The molecular structures determined by X‐ray diffraction analyses revealed distorted tetrahedral geometries around the titanium atom, and the bond distances found between the titanium and carbene carbon atoms suggest that the WCA‐NHC ligand coordinates to titanium as a strong donor ligand. The conversion of these dichloro complexes into the dimethyl derivatives is performed by reaction of **16** **a** with methyl magnesium bromide, giving [(F_5_C_6_)_3_B‐IXy]TiLMe_2_ (**16** **b**). Both types of half‐sandwich titanium(IV) complexes were used as catalysts for the ethylene polymerization in the presence of Al*i*Bu_3_ co‐catalyst, affording ultrahigh molecular weight polyethylene. Additionally, the dimethyl titanium(IV) complexes were used for the ethylene/1‐hexene co‐polymerization in the presence of MAO and the resulting polymers possessed high molecular weights with unimodal molecular weight distributions suggesting that these polymerizations proceed with an uniform catalytically active species (single site character).^[40]^

### WCA‐NHCs in Main‐Group‐Element Chemistry

**Figure 7:**
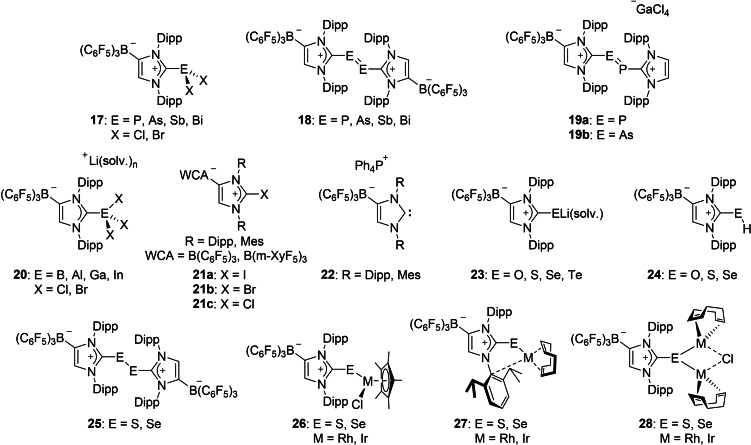
Selected examples for WCA‐NHC main group element complexes.

In the recent decades, N‐heterocyclic carbenes received considerable interest as neutral ligands for the isolation of highly reactive, low valent and low coordinate main group element compounds.[^5^, ^6^, ^7^, ^9^, ^10^] With this in mind and also following the same guiding principle of converting otherwise cationic NHC transition metal complexes into their neutral congeners with the use of WCA‐NHCs (see above), we intended to employ these anionic NHC ligands for the stabilization of cationic main‐group element fragments. The WCA‐NHCs made their first appearance in main group element chemistry in 2018, when we targeted neutral analogs of previously reported dicationic bis(NHC) stabilized diphosphenes^[41]^ and diarsenes,^[42]^ compounds which feature a double bond between the heavier pnictogen atoms. Accordingly, salt metathesis reactions between [(F_5_C_6_)_3_B‐IDipp]Li(toluene) (**6** **b**) and the pnictogen trihalides EX_3_ (E=P, As, Sb, Bi; X=Cl, Br) in toluene afforded the corresponding dihalide complexes [(F_5_C_6_)_3_B‐IDipp]EX_2_ (**17**, Figure 7), which can be regarded as an anionic NHC stabilized pnictenium fragment EX_2_^+^.^[43]^ In the solid‐state structures, short element‐arene contacts (“Menshutkin interaction”)^[44]^ are observed between the pnictogen and the *ipso*‐carbon atom of the Dipp substituent adjacent to the borate moiety, which are significantly smaller than the sum of the van der Waals radii of the involved atoms. Similar structural motifs were found in WCA‐NHC transition metal complexes, where π‐face donation was observed (see above). Upon two‐electron reduction of these dihalide complexes **17**, the corresponding diphosphene, diarsene, distibene and dibismuthene compounds **18** were obtained.[^43^, ^45^] Thereby, either elemental magnesium or 1,4‐bis(trimethylsilyl)‐1,4‐dihydropyrazine were used as the reducing agents, with the later avoiding the generation of inorganic salts, which were difficult to separate from the resulting dipnictenes. All dipnictenes exhibit the typical *trans*‐bent (*E*)‐geometry in the solid state, with E−E distances in accordance with pnictogen‐pnictogen double bonds. Once again, short element‐arene contacts were found and since earlier computational investigations from Schreiner and co‐workers found that “London dispersion decisively contributes to the thermodynamic stability of bulky NHC‐coordinated main group compounds”,^[46]^ this effect was also studied for the WCA‐NHC dihalo pnictanes **17** as well as for the dipnictenes **18**. We found that not only ligand‐element interactions, but also ligand‐ligand dispersion interactions contribute to the overall stability of the WCA‐NHC coordinated dipnictenes.^[43]^

**Figure 8:**
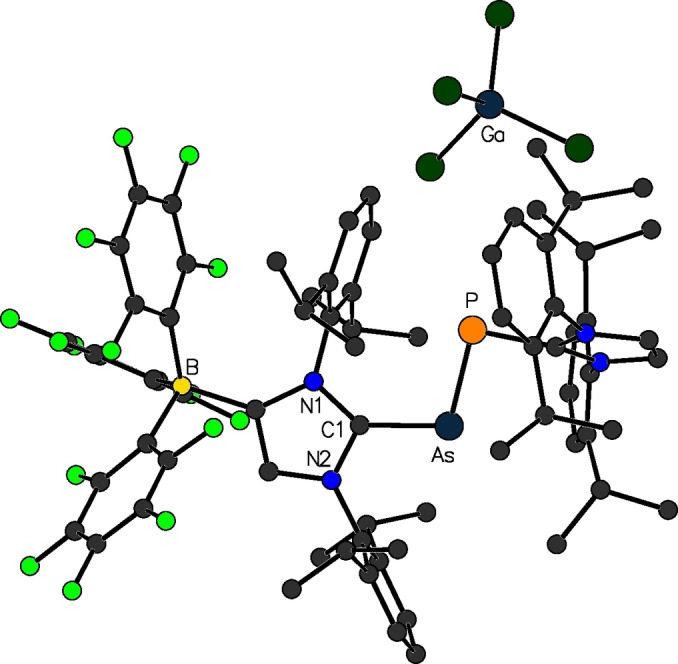
Molecular structure of [{(F_5_C_6_)_3_B‐IDipp}As=P{IDipp}][GaCl_4_] (**19** **b**).

Heteroleptic dipnictenes bearing one neutral NHC and one anionic NHC ligand were reported by our group in 2019. For their preparation, we used the so‐called modular approach,^[47]^ where the trimethylsilyl substituted NHC‐phosphinidene adduct (IDipp)PSiMe_3_ was reacted with [(F_5_C_6_)_3_B‐IDipp]ECl_2_ (**17**, E=P, As), to provide the mixed dielement species [(F_5_C_6_)_3_B‐IDipp]E(Cl)P[IDipp], in which an element‐element single bond was formed upon Me_3_SiCl elimination. A subsequent chloride abstraction reaction with GaCl_3_ converted the single bond into the desired double bond, yielding tetrachlorogallate salts [{(F_5_C_6_)_3_B‐IDipp}E=P{IDipp}][GaCl_4_] (**19** **a**: E=P; **19** **b**: E=As, Figure 8), both as orange solids after recrystallization from chlorobenzene. Alternatively, one‐electron reduction reactions can be performed with [(F_5_C_6_)_3_B‐IDipp]E(Cl)P[IDipp], giving access to neutral radical species [{(F_5_C_6_)_3_B‐IDipp}EP{IDipp}]^•^, in which the solid state‐structures revealed phosphorus‐phosphorus and phosphorus‐arsenic bond lengths that are in between the single bonds found in [(F_5_C_6_)_3_B‐IDipp]E(Cl)P[IDipp] and a double bond found in the dipnictenes **19**. Furthermore, the recorded ^31^P NMR spectra of **19**, as well as the EPR spectra for the radical species, emphasize the asymmetric coordination environments of the main group element moiety.^[48]^

In contrast to the above discussed pnictogen compounds, lithium halide elimination was not achieved during the reaction of [(F_5_C_6_)_3_B‐IDipp]Li(toluene) (**6** **b**) with group 13 element trihalides EX_3_ (E=B, Al, Ga, In; X=Cl, Br), despite our hopes that a potential intramolecular arene coordination would stabilize a vacant coordination site at the triel. In all cases, the resulting complexes [{(F_5_C_6_)_3_B‐IDipp}EX_3_]Li(solv.) (**20**) feature tetracoordinated boron, aluminum, gallium, or indium atoms in the solid‐state structures, respectively. Furthermore, the lithium atoms initiate the formation of coordination polymers, except for the indium trichloride derivative, where the lithium cation is fully solvated by four THF molecules.^[49]^

**Figure 9:**
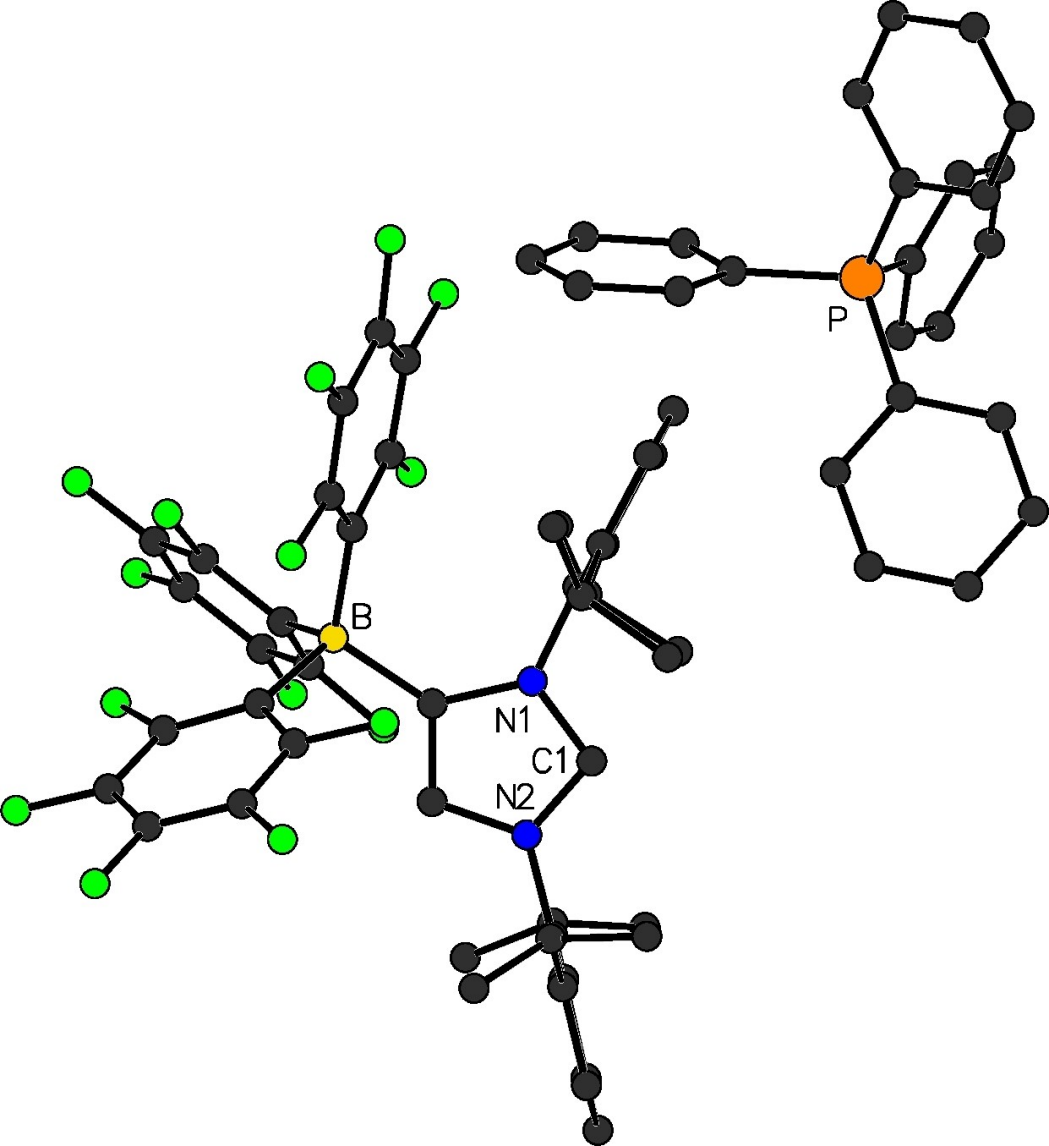
Molecular structure of [(F_5_C_6_)_3_B‐IDipp][PPh_4_] (**22**).

Halogen complexes of WCA‐NHCs were targeted by our group in 2020,^[50]^ since the concept of halogen bonding is an important issue in azolium‐based compounds, where the electrophilicity of the polarized halogen atom can be exploited to coordinate nucleophilic compounds (σ‐hole interaction). Therefore, [WCA‐NHC]Li(toluene) was reacted with iodine, bromine or CCl_4_, yielding the zwitterionic 2‐halogenoimidazolium borates [WCA‐NHC]X (**21** **a**–**c**: X=I, Br, Cl; WCA=B(C_6_F_5_)_3_, B(m‐XyF_6_)_3_; NHC=IDipp, IMes). The iodine derivative [(F_5_C_6_)_3_B‐IDipp]I was able to coordinate solvent molecules such as toluene or chlorobenzene via σ‐hole‐π‐interactions. Acetonitrile, THF and trimethylamine oxide molecules can act as a σ donor towards the σ‐hole at the iodine atom and additionally, hypervalent bis(carbene)iodine(I) complexes of the type [{(F_5_C_6_)_3_B‐IDipp}I(NHC)] and [{(F_5_C_6_)_3_B‐IDipp}I{(F_5_C_6_)_3_B‐NHC}][PPh_4_] (NHC=IDipp, IMes) were also established. The latter compound was synthesized from the reaction of [(F_5_C_6_)_3_B‐IDipp]I with [(F_5_C_6_)_3_B‐NHC][PPh_4_] (**22**, Figure 9), compounds which we regard as important synthons for the “free” anionic N‐heterocyclic carbene. The assumption that London dispersion interactions are important for the stability of such complexes was once again confirmed by computational investigations and a quantum theory of atoms in molecules (QTAIM) analysis for the heteroleptic complex [{(F_5_C_6_)_3_B‐IDipp}I(IDipp)] revealed that the anionic carbenes WCA‐NHC act as stronger donors than their neutral NHC congeners.^[50]^

In addition to N‐heterocyclic carbenes, their main group element adducts were also widely used as ligands in transition metal chemistry.^[10]^ For instance, neutral compounds of the type (NHC)E (E=O, S, Se, Te), which can be considered as urea derivatives, were among the first isolated NHC p‐block element species. However, compared to NHC chemistry, comparatively few transition metal complexes bearing imidazolin‐2‐thiones, ‐selones and ‐tellones were reported in recent the literature. Adapting our strategy of converting neutral NHC ligands into anionic derivatives (see above), we have demonstrated in 2020 the isolation of chalcogen complexes bearing anionic N‐heterocyclic carbenes.^[51]^ The imidazolin‐2‐one and ‐thione adducts, containing a B(C_6_F_5_)_3_ moiety in the heterocyclic backbone were prepared from the deprotonation reaction of (IDipp)O or (IDipp)S with *n*BuLi at room temperature in toluene, respectively. The borane was subsequently added to these lithiated species, providing the lithium salts [{(F_5_C_6_)_3_B‐IDipp}E]Li(solv.) (**23**; E=O, S). This synthetic protocol could not be extended for the heavier congeners selenium and tellurium, since only the formation of the “normal” carbene‐borane adduct (IDipp)B(C_6_F_5_)_3_ could be observed. Instead, the direct reaction of [(F_5_C_6_)_3_B‐IDipp]Li(toluene) (**6** **b**) with elemental selenium or tellurium powder furnished the desired complexes [{(F_5_C_6_)_3_B‐IDipp}E]Li(solv.) (**23**; E=Se, Te). Initially, we studied these new ligands regarding their behavior towards protonation or oxidation. The protonation with HCl in Et_2_O afforded the species [(F_5_C_6_)_3_B‐IDipp]EH (**24**; E=O, S, Se). One‐electron oxidations of **23** with ferrocenium hexafluorophosphate provided the corresponding disulfide and diselenide compounds [(F_5_C_6_)_3_B‐IDipp]_2_E_2_ (**25**; E=S, Se).^[51]^

One common method to determine the π‐accepting properties of N‐heterocyclic carbenes is to measure the ^77^Se NMR shifts of the corresponding (NHC)Se adducts.^[52]^ With a reliable method to access to the selenium adducts of WCA‐NHCs in hand, we were interested to assess the π‐accepting capabilities of our ligands in a similar fashion. Therefore, several [(WCA‐NHC)Se]Y (WCA=B(C_6_F_5_)_3_, Al(C_6_F_5_)_3_, Ga(C_6_F_5_)_3_; NHC=IDipp, IMes; Y=Li(solv.), PPh_4_) adducts were prepared to study the influence of the WCA backbone and nitrogen‐substituent variation. One important conclusion of these investigations was that the counter cation does not exert any influence on the selenium atom, if it is fully separated from the anionic carbene moiety in solution. Furthermore, we found that the ^77^Se NMR shifts for the (WCA‐NHC)Se adducts are found to be in the same range as for neutral congeners (IDipp)Se and (IMes)Se, suggesting only marginal influence of the anionic moiety at the 4‐position of the imidazole ring on the π‐acidity of the NHC.^[23]^

**Figure 10:**
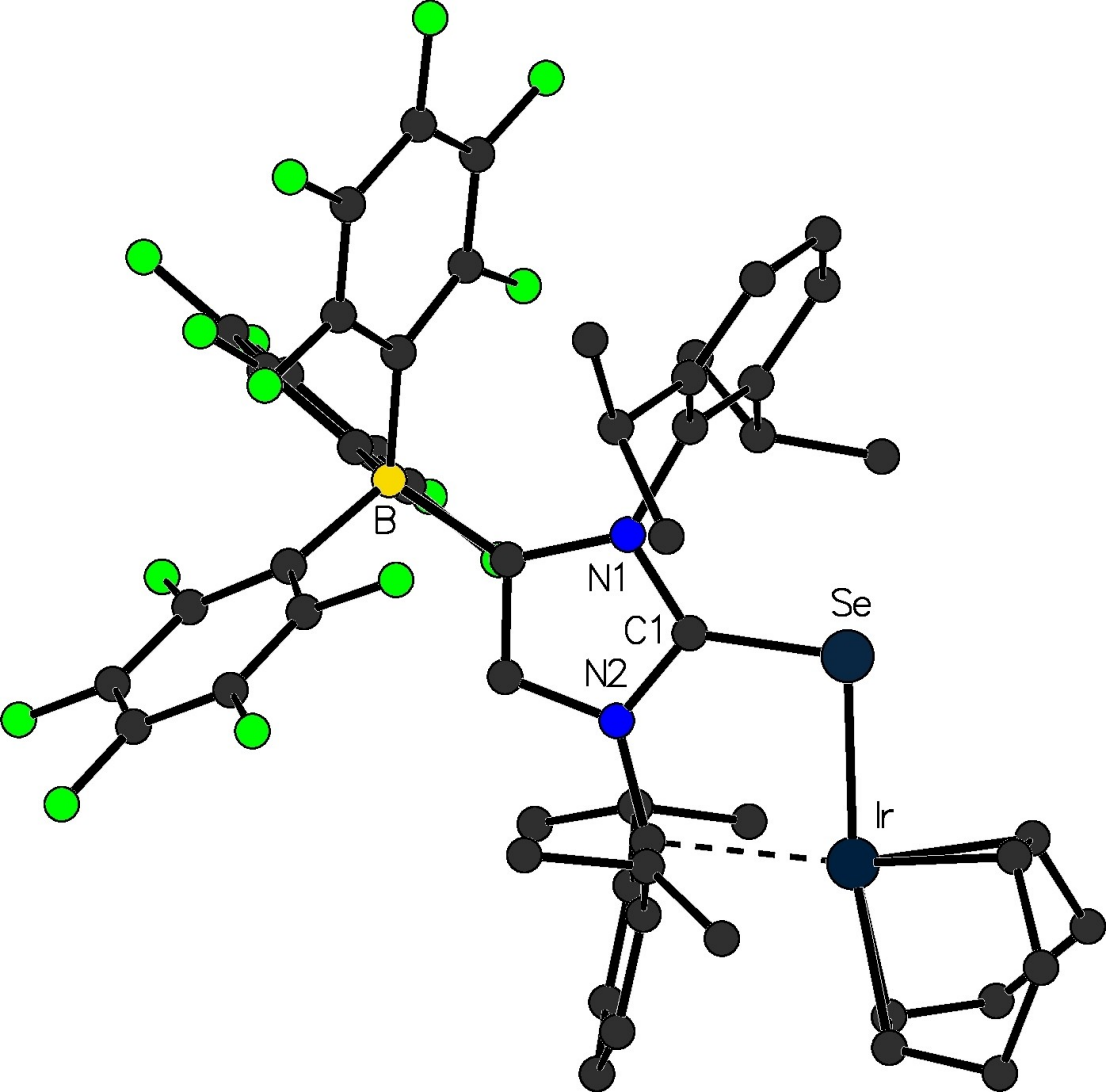
Molecular structure of [{(F_5_C_6_)_3_B‐IDipp}Se]Ir(COD) (**27**).

Our interest in these anionic imidazolin‐2‐thione and ‐selone ligands are based on the long‐term goal to provide a new ligand system for the preparation of numerous neutral transition metal complexes, which would be otherwise cationic when regular thione or selone ligands were used. In addition to the use of the lithium salts [{(F_5_C_6_)_3_B‐IDipp}E]Li(solv.) (**23**; E=S, Se) as anionic thione or selone transfer reagents, the corresponding trimethyl silyl derivatives [(F_5_C_6_)_3_B‐IDipp]ESiMe_3_ are also suitable for the reaction with transition metal halide precursors,^[53]^ in a similar fashion as performed with the trimethyl silyl NHC‐phosphinidenide (NHC)PSiMe_3_.^[54]^ These compounds are straightforwardly available from the reaction of **23** with Me_3_SiCl, resulting in the elimination of lithium chloride and the attachment of the silyl group to the chalcogen atom. With both these reagents, several rhodium and iridium complexes were established, which have an identical total charge as the corresponding NHC‐phosphinidenide complexes [{(IDipp)P}ML_n_], and therefore, a comparison of phosphorus‐metal and chalcogen‐metal bonds was possible, while preserving the overall structural and electronic properties. For instance, the half sandwich complexes [{(F_5_C_6_)_3_B‐IDipp}E]MCl(*η* ^5^‐C_5_Me_5_) (**26**; E=S, Se; M=Rh^III^, Ir^III^) were isolated via the reaction of [(F_5_C_6_)_3_B‐IDipp]ESiMe_3_ with 0.5 equiv. of the dimers [(*η* ^5^‐C_5_Me_5_)MCl_2_]_2_. Interestingly, with the rhodium(I) and iridium(I) cyclooctadiene substrates [M(COD)Cl]_2_, either the monometallic complexes [{(F_5_C_6_)_3_B‐IDipp}E]M(COD) (**27**, Figure 10) or bimetallic complexes [{(F_5_C_6_)_3_B‐IDipp}E]M_2_(COD)_2_(*μ* ^2^‐Cl) (**28**; E=S, Se; M=Rh^I^, Ir^I^) are obtained, depending on the stoichiometry used during the reaction. One notable structural anomaly is observed in the solid‐state structures of the monometallic complexes **27**, where for the first‐time metal‐arene coordination is present towards the nitrogen‐substituent opposite to the borate moiety. In contrast, all other isolated transition metal complexes containing a WCA‐NHC ligand, display this interaction towards the arene adjacent to the B(C_6_F_5_)_3_ group (see above).

Computational investigations revealed that the experimentally observed *anti*‐orientation of the borate moiety with respect to the iridium atom is slightly energetically favored. In contrast to the corresponding NHC‐phosphinidenide complexes, where significant metal‐phosphorus π‐bond contribution was consistently found, only a negligible degree of chalcogen‐metal π‐bonding is present in these chalcogen complexes.

### What Lies Ahead?

Important fundamental questions for instance regarding the σ‐donor capabilities^[20]^ or the influence of the backbone and nitrogen‐substituents on the π‐acceptor strengths of WCA‐NHCs^[23]^ as well as the impact of the nitrogen‐substituents on the overall stability of complexes via π‐face donation[^20^, ^36^, ^37^] or London Dispersion stabilization[^43^, ^50^] have been addressed by our group in previous contributions. This provided understanding why the coordination of the WCA‐NHC ligand to certain metals is beneficial to their catalytic applications, for example, in gold(I) catalysis, without the need for the activation with silver(I) salts. Despite these achievements, there are still unknown factors that need to be explored. For instance, how can we facilitate lithium halide elimination more reliably? Do the sodium and potassium derivatives **6** **h**–**6** **j** (Scheme 1) behave differently in salt metathesis reactions than their lithium counterparts? Do we still observe π‐face donation interactions in complexes, if we attach smaller borate moieties in the imidazole backbone? What happens when the heterocycle itself is modified?

Covering early to late transition metals, WCA‐NHCs have demonstrated on several occasions their remarkable ability to provide overall neutral complexes, some of which are capable to facilitate catalytic processes. Initial investigations regarding the isolation of group 8 complexes, targeting for instance ruthenium (see above), unravelled certain difficulties to transfer the anionic NHC moiety to these transition metals. However, complexes with group 6 or 8 metals might be of considerable interest, as we demonstrated repeatedly that complexes containing WCA‐NHC ligands display in many cases superior catalytic activity, compared to their cationic NHC counterparts. Iron and ruthenium complexes, potentially potent for hydroelementation reactions, or the influence of WCA‐NHCs on the catalytic activity of molybdenum and tungsten complexes regarding alkyne metathesis reactions have not yet been explored. Moreover, the chemistry of the f‐block elements with anionic NHCs also remained uncharted so far.^[55]^

The main group element chemistry involving WCA‐NHCs has witnessed considerable progress in the last five years. Numerous complexes such as **17**, **20** and **23** (Figure 7) were isolated, allowing for the extensive expansion of a modular approach,^[47]^ to isolate a plethora of mixed dielement species, stabilized by WCA‐NHCs alone or in combination with neutral NHCs. Furthermore, the anionic thione and selone species bear great potential as ancillary ligands in coordination chemistry. For example, considering the affinity of sulfur ligands to, for example, iron and gold, one might witness the application of WCA‐NHC thione complexes for bioinorganic applications in the future.

## Conflict of interest

The authors declare no conflict of interest.

## Biographical Information

*Luong Phong Ho, a second‐generation immigrant with parents originating from Vietnam, was born in 1994 in Wolfsburg, Germany. He studied chemistry at the Technische Universität Braunschweig, where he obtained his Bachelor and Master degree in 2015 and 2017, respectively. In 2017 he started his Ph.D. studies under the supervision of Prof. Dr. Matthias Tamm, which he has successfully completed in October 2020. His research revolved around main group element complexes of anionic N‐heterocyclic carbenes carrying weakly coordinating anions*.

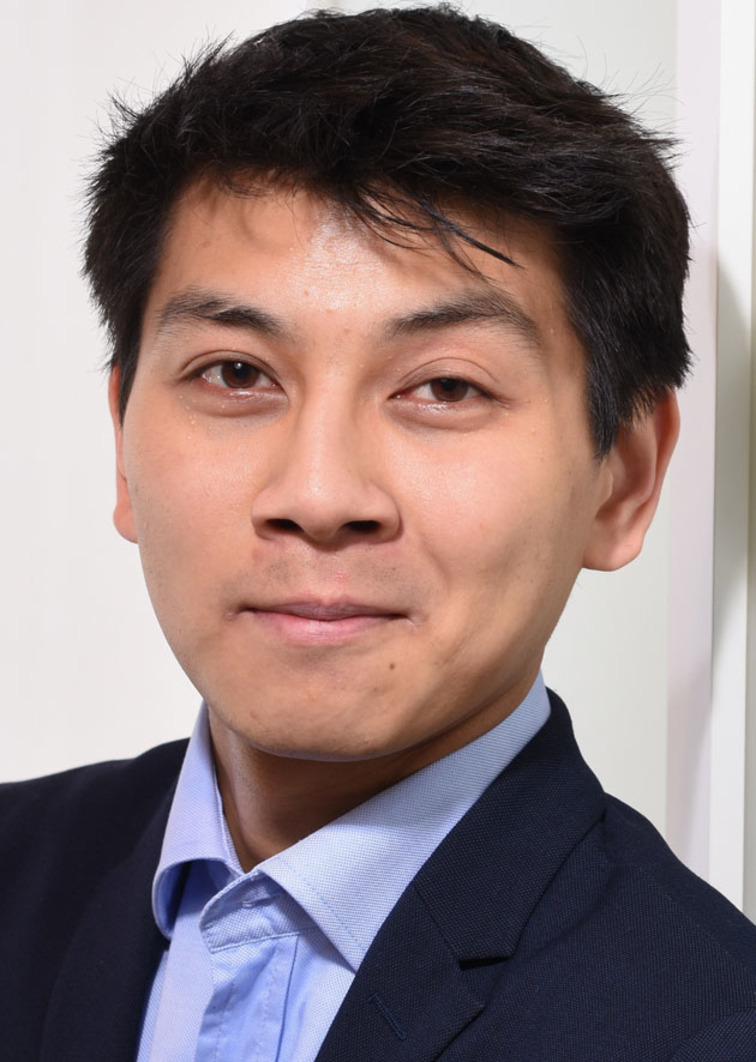

## Biographical Information

*Matthias Tamm studied chemistry at the Technische Universität Berlin, where he received his PhD in 1992 under the supervision of F. Ekkehardt Hahn. After spending one year as a Visiting Research Scientist at DuPont Central Research and Development, Experimental Station, Wilmington, USA with Anthony J. Arduengo, III, he returned to Germany in 1994 to complete his Habilitation. Tamm was appointed to Privatdozent at the Westfälische Wilhelms‐Universität Münster in 1999 and held a temporary professorship at the Technische Universität München from 2002 to 2005. He moved to the Technische Universität Braunschweig in 2005 to become full professor of inorganic chemistry*.

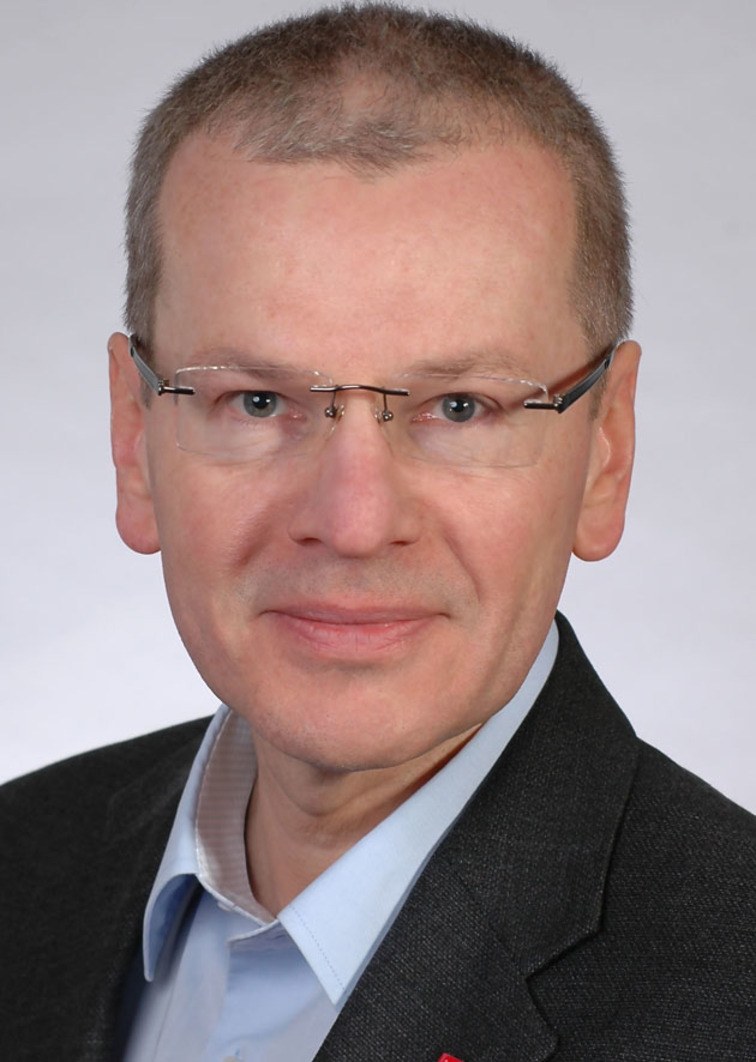

## References

[1] A. J. Arduengo , R. L. Harlow , M. Kline , J. Am. Chem. Soc. 1991, 113, 361.

[2] D. Bourissou , O. Guerret , F. P. Gabbaï , G. Bertrand , Chem. Rev. 2000, 100, 39. 11749234 10.1021/cr940472u

[3a] F. E. Hahn , M. C. Jahnke , Angew. Chem. Int. Ed. 2008, 47, 3122; 10.1002/anie.200703883 18398856

[3b] M. N. Hopkinson , C. Richter , M. Schedler , F. Glorius , Nature 2014, 510, 485; 24965649 10.1038/nature13384

[3c] W. A. Herrmann , C. Köcher , Angew. Chem. Int. Ed. 1997, 36, 2162.

[4a] J. C. Garrison , W. J. Youngs , Chem. Rev. 2005, 105, 3978; 16277368 10.1021/cr050004s

[4b] R. Singh , S. P. Nolan , Annu. Rep. Prog. Chem. Sect. B 2006, 102, 168;

[4c] H. G. Raubenheimer , S. Cronje , Chem. Soc. Rev. 2008, 37, 1998; 18762843 10.1039/b708636a

[4d] P. de Frémont , N. Marion , S. P. Nolan , Coord. Chem. Rev. 2009, 253, 862;

[4e] S. Díez-González , N. Marion , S. P. Nolan , Chem. Rev. 2009, 109, 3612; 19588961 10.1021/cr900074m

[4f] George C. Fortman , Steven P. Nolan , Chem. Soc. Rev. 2011, 40, 5151; 21731956 10.1039/c1cs15088j

[4g] R. Visbal , M. C. Gimeno , Chem. Soc. Rev. 2014, 43, 3551; 24604135 10.1039/c3cs60466g

[4h] D. Zhang , G. Zi , Chem. Soc. Rev. 2015, 44, 1898; 25608933 10.1039/c4cs00441h

[4i] C. A. Smith , M. R. Narouz , P. A. Lummis , I. Singh , A. Nazemi , C.-H. Li , C. M. Crudden , Chem. Rev. 2019, 119, 4986; 30938514 10.1021/acs.chemrev.8b00514

[4j] A. A. Danopoulos , T. Simler , P. Braunstein , Chem. Rev. 2019, 119, 3730. 30843688 10.1021/acs.chemrev.8b00505

[5] Y. Wang , G. H. Robinson , Inorg. Chem. 2011, 50, 12326. 21634365 10.1021/ic200675u

[6] Y. Wang , G. H. Robinson , Dalton Trans. 2012, 41, 337. 21904737 10.1039/c1dt11165e

[7] Y. Wang , G. H. Robinson , Inorg. Chem. 2014, 53, 11815. 25343222 10.1021/ic502231m

[8] K. Schwedtmann , G. Zanoni , J. J. Weigand , Chem. Asian J. 2018, 13, 1388. 29573181 10.1002/asia.201800199

[9] V. Nesterov , D. Reiter , P. Bag , P. Frisch , R. Holzner , A. Porzelt , S. Inoue , Chem. Rev. 2018, 118, 9678. 29969239 10.1021/acs.chemrev.8b00079

[10] A. Doddi , M. Peters , M. Tamm , Chem. Rev. 2019, 119, 6994. 30983327 10.1021/acs.chemrev.8b00791

[11a] D. Munz , Organometallics 2018, 37, 275;

[11b] T. Dröge , F. Glorius , Angew. Chem. Int. Ed. 2010, 49, 6940; 10.1002/anie.201001865 20715233

[11c] D. J. Nelson , S. P. Nolan , Chem. Soc. Rev. 2013, 42, 6723; 23788114 10.1039/c3cs60146c

[11d] H. V. Huynh , Chem. Rev. 2018, 118, 9457. 29601194 10.1021/acs.chemrev.8b00067

[12] A. Nasr , A. Winkler , M. Tamm , Coord. Chem. Rev. 2016, 316, 68.

[13a] B. Royo , E. Peris , Eur. J. Inorg. Chem. 2012, 1309;

[13b] S. T. Liddle , I. S. Edworthy , P. L. Arnold , Chem. Soc. Rev. 2007, 36, 1732. 18213982 10.1039/b611548a

[14a] S. P. Nolan , Acc. Chem. Res. 2011, 44, 91; 21028871 10.1021/ar1000764

[14b] N. Marion , S. P. Nolan , Chem. Soc. Rev. 2008, 37, 1776. 18762827 10.1039/b711132k

[15] S. Kronig , E. Theuergarten , C. G. Daniliuc , P. G. Jones , M. Tamm , Angew. Chem. Int. Ed. 2012, 51, 3240. 10.1002/anie.201108813 22337636

[16a] M. Stradiotto , K. D. Hesp , R. J. Lundgren , Angew. Chem. Int. Ed. 2010, 49, 494; 10.1002/anie.200904093 19998395

[16b] R. Chauvin , Eur. J. Inorg. Chem. 2000, 577.

[17] D. Holschumacher , T. Bannenberg , C. G. Hrib , P. G. Jones , M. Tamm , Angew. Chem. Int. Ed. 2008, 47, 7428. 10.1002/anie.200802705 18666192

[18] Y. Wang , Y. Xie , M. Y. Abraham , P. Wei , H. F. Schaefer , P. v. R. Schleyer , G. H. Robinson , J. Am. Chem. Soc. 2010, 132, 14370. 20863065 10.1021/ja106631r

[19a] M. Uzelac , A. Hernán-Gómez , D. R. Armstrong , A. R. Kennedy , E. Hevia , Chem. Sci. 2015, 6, 5719; 29910864 10.1039/c5sc02086g PMC5975842

[19b] M. Uzelac , A. R. Kennedy , A. Hernán-Gómez , M. Á. Fuentes , E. Hevia , Z. Anorg. Allg. Chem. 2016, 642, 1241.

[20] E. L. Kolychev , S. Kronig , K. Brandhorst , M. Freytag , P. G. Jones , M. Tamm , J. Am. Chem. Soc. 2013, 135, 12448. 23883399 10.1021/ja406529c

[21] A. Igarashi , E. L. Kolychev , M. Tamm , K. Nomura , Organometallics 2016, 35, 1778.

[22] J. Frosch , L. Körner , M. Koneczny , M. Tamm , Z. Anorg. Allg. Chem. 2021, 647, 998.

[23] L. P. Ho , M. Koneczny , T. Bannenberg , M. Tamm , Inorg. Chem. 2021, 60, 9019. 34042436 10.1021/acs.inorgchem.1c01004

[24] N. Phillips , R. Tirfoin , S. Aldridge , Dalton Trans. 2014, 43, 15279. 25198297 10.1039/c4dt02662d

[25] H. Niu , R. J. Mangan , A. V. Protchenko , N. Phillips , W. Unkrig , C. Friedmann , E. L. Kolychev , R. Tirfoin , J. Hicks , S. Aldridge , Dalton Trans. 2018, 47, 7445. 29782026 10.1039/c8dt01661e

[26] A. El-Hellani , V. Lavallo , Angew. Chem. Int. Ed. 2014, 53, 4489. 10.1002/anie.201402445 24664969

[27] M. J. Asay , S. P. Fisher , S. E. Lee , F. S. Tham , D. Borchardt , V. Lavallo , Chem. Commun. 2015, 51, 5359. 10.1039/c4cc08267b 25387660

[28] J. Estrada , V. Lavallo , Angew. Chem. Int. Ed. 2017, 129, 10038.

[29a] F. Medici , G. Gontard , E. Derat , G. Lemière , L. Fensterbank , Organometallics 2018, 37, 517, 10.1021/acs.organomet.7b00838;

[29b] T. Deis , F. Medici , A. Poussard-Schulz , G. Lemière , L. Fensterbank , J. Organomet. Chem. 2021, 956, 122120, 10.1016/j.jorganchem.2021.122120.

[30] K. C. Weerasiri , D. Chen , D. I. Wozniak , G. E. Dobereiner , Adv. Synth. Catal. 2016, 358, 4106.

[31] S. Planer , J. Frosch , M. Koneczny , D. Trzybiński , K. Woźniak , K. Grela , M. Tamm , Chem. Eur. J. 2021, 27, 15217. 34342923 10.1002/chem.202102553 PMC8597159

[32] S. P. Fisher , A. El-Hellani , F. S. Tham , V. Lavallo , Dalton Trans. 2016, 45, 9762. 26922968 10.1039/c6dt00551a

[33] S. P. Fisher , S. G. McArthur , V. Tej , S. E. Lee , A. L. Chan , I. Banda , A. Gregory , K. Berkley , C. Tsay , A. L. Rheingold , G. Guisado-Barrios , V. Lavallo , J. Am. Chem. Soc. 2020, 142, 251. 31804820 10.1021/jacs.9b10234

[34] J. Holmes , R. J. Kearsey , K. A. Paske , F. N. Singer , S. Atallah , C. M. Pask , R. M. Phillips , C. E. Willans , Organometallics 2019, 38, 2530.

[35] M. Koneczny , L. Phong Ho , A. Nasr , M. Freytag , P. G. Jones , M. Tamm , Adv. Synth. Catal. 2020, 362, 3857.

[36] A. Winkler , K. Brandhorst , M. Freytag , P. G. Jones , M. Tamm , Organometallics 2016, 35, 1160.

[37] J. Frosch , M. Freytag , P. G. Jones , M. Tamm , J. Organomet. Chem. 2020, 918, 121311.

[38] K. Nomura , G. Nagai , I. Izawa , T. Mitsudome , M. Tamm , S. Yamazoe , ACS Omega 2019, 4, 18833. 31737845 10.1021/acsomega.9b02828 PMC6854829

[39] G. Nagai , T. Mitsudome , K. Tsutsumi , S. Sueki , T. Ina , M. Tamm , K. Nomura , J. Jpn. Petrol. Inst. 2017, 60, 256.

[40] K. Nomura , G. Nagai , A. Nasr , K. Tsutsumi , Y. Kawamoto , K. Koide , M. Tamm , Organometallics 2019, 38, 3233.

[41] O. Back , B. Donnadieu , P. Parameswaran , G. Frenking , G. Bertrand , Nat. Chem. 2010, 2, 369. 20414236 10.1038/nchem.617

[42] M. Y. Abraham , Y. Wang , Y. Xie , R. J. Gilliard , P. Wei , B. J. Vaccaro , M. K. Johnson , H. F. Schaefer , P. v. R. Schleyer , G. H. Robinson , J. Am. Chem. Soc. 2013, 135, 2486. 23363453 10.1021/ja400219d

[43] L. P. Ho , A. Nasr , P. G. Jones , A. Altun , F. Neese , G. Bistoni , M. Tamm , Chem. Eur. J. 2018, 24, 18922. 30357989 10.1002/chem.201804714

[44] M. Krasowska , W. B. Schneider , M. Mehring , A. A. Auer , Chem. Eur. J. 2018, 24, 10238. 10.1002/chem.201801758 29718544

[45] L. P. Ho , M. Tamm , Dalton Trans. 2021, 50, 1202. 33480906 10.1039/d1dt00140j

[46] J. P. Wagner , P. R. Schreiner , J. Chem. Theory Comput. 2016, 12, 231. 26606127 10.1021/acs.jctc.5b01100

[47] A. Doddi , D. Bockfeld , M.-K. Zaretzke , C. Kleeberg , T. Bannenberg , M. Tamm , Dalton Trans. 2017, 46, 15859. 29114655 10.1039/c7dt03436a

[48] L. P. Ho , M.-K. Zaretzke , T. Bannenberg , M. Tamm , Chem. Commun. 2019, 55, 10709. 10.1039/c9cc05739k 31429453

[49] L. P. Ho , L. Anders , M. Tamm , Chem. Asian J. 2020, 15, 845–851. 32011782 10.1002/asia.201901774 PMC7154526

[50] J. Frosch , M. Koneczny , T. Bannenberg , M. Tamm , Chem. Eur. J. 2020, 4349. 33094865 10.1002/chem.202004418 PMC7986712

[51] L. P. Ho , L. Körner , T. Bannenberg , M. Tamm , Dalton Trans. 2020, 49, 13207. 32785308 10.1039/d0dt02392b

[52] A. Liske , K. Verlinden , H. Buhl , K. Schaper , C. Ganter , Organometallics 2013, 32, 5269.

[53] L. P. Ho , A. Neitzel , T. Bannenberg , M. Tamm , Chem. Eur. J. 2022, 28, e202104139. 34878696 10.1002/chem.202104139 PMC9305287

[54] A. Doddi , D. Bockfeld , T. Bannenberg , P. G. Jones , M. Tamm , Angew. Chem. Int. Ed. 2014, 53, 13568. 10.1002/anie.201408354 25287885

[55] P. L. Arnold , I. J. Casely , Chem. Rev. 2009, 109, 3599. 19358527 10.1021/cr8005203

